# C-KIT-positive undifferentiated tumor of the liver: A case report

**DOI:** 10.3892/ol.2014.2324

**Published:** 2014-07-08

**Authors:** HYUN HEE CHU, BAIK HWAN CHO, JI SOO SONG, KYUNG MI KIM, WOO SUNG MOON

**Affiliations:** 1 Department of Pathology, Chonbuk National University, Medical School, Research Institute for Endocrine Sciences and Research Institute of Clinical Medicine, Jeonju 561-756, Republic of Korea; 2 Department of Surgery, Chonbuk National University, Medical School, Research Institute for Endocrine Sciences and Research Institute of Clinical Medicine, Jeonju 561-756, Republic of Korea; 3 Department of Radiology, Chonbuk National University, Medical School, Research Institute for Endocrine Sciences and Research Institute of Clinical Medicine, Jeonju 561-756, Republic of Korea; 4 Department of Pathology, Samsung Medical Center, Sungkyunkwan University School of Medicine, Seoul 135-710, Republic of Korea

**Keywords:** C-KIT, stem cell, liver

## Abstract

With recent advances in cancer stem cell analysis, it has been postulated that the transformation of hepatic stem and progenitor cells underlies the development of certain liver cancers. Human C-KIT is a transmembrane type III receptor protein with intrinsic tyrosine kinase activity that has been proposed as a marker for human embryonic stem cells. In addition, human C-KIT functions in maintaining the undifferentiated state of stem cells, and has been identified as a marker for human hematopoietic and hepatic stem/progenitor cells. The present study identified an unusual case of a C-KIT-positive hepatic tumor with an undifferentiated stem cell phenotype distinct from existing descriptions of liver tumors. A 69-year-old male with Ampulla of Vater (AoV) cancer was admitted to the hospital for the treatment of a hepatic mass that was incidentally detected during evaluation of AoV cancer. Microscopically, the hepatic tumor was composed of solidly packed small, round and uniform undifferentiated cells, which resembled that of a small-blue-round-cell tumor. The immunophenotype of neoplastic cells (C-KIT^+^/EpCAM^+^/E-cadherin^+^/keratin 7^−^/keratin 19^−^/α-fetoprotein^−^/albumin^−^) supported primitive stem cell features with no hepatic or biliary phenotypes. Polymerase chain reaction and direct DNA sequencing revealed no C-KIT mutations. It is suggested that this tumor may have originated from transformed C-KIT^+^/EpCAM^+^/E-cadherin^+^ cells, which are more primitive and undifferentiated than bipotential hepatic progenitor cells.

## Introduction

The two general models of carcinogenesis postulate clonal evolution and growth through cancer stem cells (CSCs). According to the CSC model, a single CSC gives rise to a hierarchical organization within a tumor (1,2). Recent studies of hepatic tumors have focused on CSCs, including the detection of CSCs in cancer, development of CSC markers and therapeutic targeting of CSCs (2,3). Primary hepatic malignant tumors, including a subset of hepatocellular carcinomas (HCCs), cholangiocarcinoma (CCs), combined hepatocellular carcinoma and cholangiocarcinoma (c-HCC-CCs), and hepatoblastomas (HBs) are considered to originate from hepatic stem cells or progenitor cells (4–9). Despite recent efforts to understand the contribution of CSCs to hepatocarcinogenesis, it remains unclear whether CSCs are derived from resident liver stem/progenitor cells, bone marrow or differentiated mature cells that have undergone a de-differentiation or a trans-differentiation process (2,3). Likewise, there are currently no specific surface markers for identifying the origin of hepatic CSCs. Hepatic stem/progenitor cells share surface markers associated with hematopoietic stem/progenitor cells including CD34, CD90 and C-KIT (CD117), while hepatic stem/progenitor cells can be distinguished from hematopoietic stem/progenitor cells due to the expression of hepatic and biliary markers such as albumin, α-fetoprotein (AFP), keratin 18 and keratin 19 (2–4,10–12).

Human C-KIT is a transmembrane type III receptor protein with intrinsic tyrosine kinase activity that transduces growth regulatory signals across the plasma membrane (11). The expression of C-KIT in human embryonic stem cells is greater than the expression observed in differentiated cells. It has therefore been proposed that C-KIT may be a useful marker for human embryonic stem cells and a central protein in maintaining their undifferentiated state (11,12). The present study describes the case of an unusual C-KIT-positive hepatic tumor with an undifferentiated stem cell phenotype distinct from known types of liver tumors.

## Case report

### Clinical presentation

**Figure 1:**
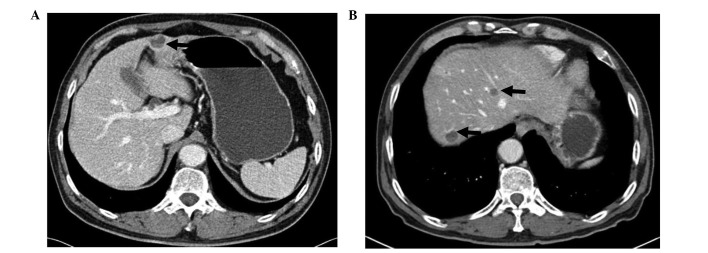
(A) A liver mass ~2 cm in size with heterogeneous internal attenuation was seen by contrast-enhanced abdominal CT (arrow). (B) Follow-up contrast-enhanced abdominal CT showed multiple newly developed liver masses with heterogeneous internal attenuation similar to the initially detected liver mass (arrows). CT, computed tomography.

A 69-year-old male was diagnosed with Ampulla of Vater (AoV) cancer in a local clinic, and was referred to the Department of Surgery at the Chonbuk National University Hospital (Jeonju, Korea) for surgical treatment. During the evaluation of AoV cancer, an abdominal computed tomography (CT) scan incidentally detected a solitary 2.0-cm-sized hepatic nodule in the subcapsular area of segment 4 (Fig. 1A). There was no history of hepatitis B or C, and the serum α-fetoprotein (AFP) levels were normal. During AoV cancer surgery, an hepatic mass excision was performed. The pathologic diagnosis was AoV cancer with a well-differentiated tubular adenocarcinoma confined to the submucosa (pT1). Immunohistochemical analysis did not detect C-KIT-positive tumor cells in the AoV cancer. Patient provided written informed consent.

### Pathological findings

**Figure 2:**
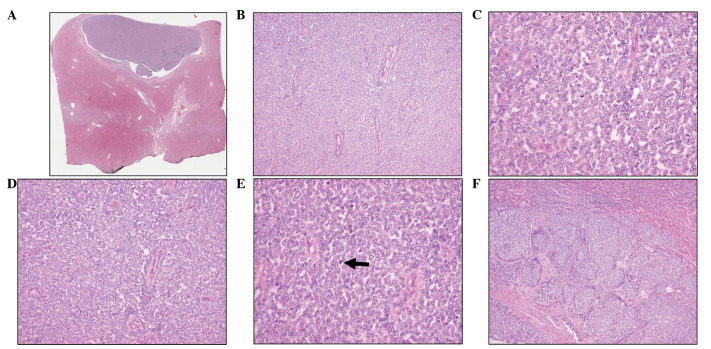
(A and B) A solid sheet comprised of undifferentiated small and uniform cells (stain, hematoxylin and eosin; magnification, A: scanning view and B: ×100). (C) Individual cells had a round or ovoid nuclei with distinct nuclear membranes and small nucleoli (stain, hematoxylin and eosin; magnification, ×400). (D) Tumor cells were arranged preferentially around hyalinized blood vessels featuring perivascular pseudorosettes (stain, hematoxylin and eosin; magnification, ×200). (E) The number of mitotic figures (arrow) was low, as compared with the immature appearance of neoplastic cells (stain, hematoxylin and eosin; magnification, ×400). (F) A nested pattern of infiltration of tumor cells into the capsule and adjacent liver tissue was observed (stain, hematoxylin and eosin; magnification, ×100).

**Figure 3:**
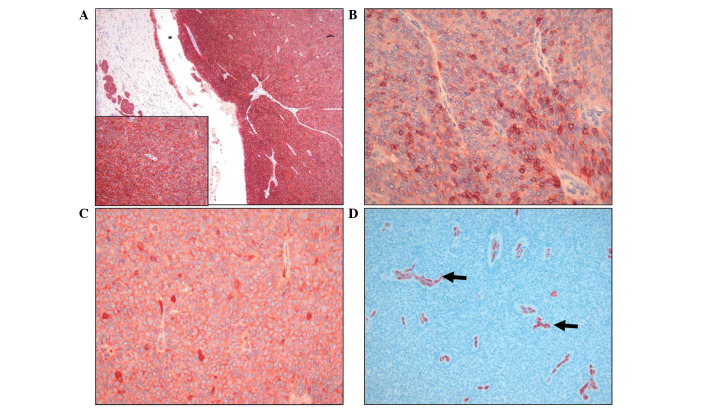
Results of immunohistochemistry. (A) Strong expression of C-KIT in tumor cells (magnification, ×100; insert, ×400). (B) Approximately 30% of tumor cells exhibited immunoreactivity for EpCAM (magnification, ×400). (C) The majority of tumor cells revealed strong expression of E-cadherin (magnification, ×400). (D) There was an absence of CD34 reactivity in tumor cells. Strong expression of CD34 in the endothelial cells of the blood vessels (arrows) was noted (magnification, ×200).

On gross examination, the hepatic tumor was well-encased in a fibrous capsule with cystic changes. The cut surface of the mass was solid and gray-white. Microscopically, the tumor was composed of solidly packed small, round, uniform cells (Fig. 2A and B). The individual cells had round or ovoid nuclei measuring 10–15 μm in diameter. The nuclear membrane was distinct, and showed inconspicuous or small nucleoli (Fig. 2C). The cytoplasm was poorly defined and scant, with pale staining. The tumor was richly vascular, and the tumor cells were often observed to be arranged preferentially around hyalinized blood vessels featuring perivascular pseudorosettes (Fig. 2D). The number of mitotic figures was not high (1–2/10 HFPs), contrasting with the immature appearance of the neoplastic cells (Fig. 2E). The tumor cells had infiltrated into the capsule and adjacent liver tissue in a nested pattern, suggesting a potential for malignancy (Fig. 2F). Immunohistochemical staining showed that the tumor cells were positive for the hepatic stem cell markers C-KIT and epithelial cell adhesion molecule (EpCAM), and the epithelial cell markers epithelial membrane antigen (EMA), E-cadherin and β-catenin, and negative for hepatic and biliary markers (Table I and Fig. 3). The tumor was evaluated for *C-KIT* mutations in exons 9, 11, 13 and 17 by polymerase chain reaction and direct DNA sequencing; however, no *C-KIT* mutations were detected. No other tumors were identified by systemic examinations. A follow-up CT scan 30 months after surgery revealed four newly developed liver masses with heterogeneous internal attenuation similar to that of the initially detected liver mass (Fig. 1B). A biopsy from the recurrent tumor showed the same histopathological and immunohistochemical findings of the primary tumor. The general condition of the patient was good and they refused further treatment.

## Discussion

Hepatic stem cells are considered as a heterogeneous population with a potential hierarchical organization and various degrees of differentiation (10,13). CSCs in liver cancer can also be highly heterogeneous, and the status of CSC marker expression may be a key determinant of cancer phenotype with respect to both the tumorigenic potential and chemosensitivity (10,14). Accumulating evidence has suggested that HCC, CC, c-HCC-CC and HBs are histologically heterogeneous and contain a subset of cells that express variable stem cell markers (4–9). Histologically, the tumors identified in this study consisted of uniformly small, solidly packed undifferentiated cells with a scant cytoplasm. These cells were subsequently found to express a stem cell immunophenotype. The individual cellular findings were consistent with the typical morphological features of tumor cells in putative hepatic stem cell tumors, such as c-HCC-CC and HBs; however, the tumor also exhibited several unusual features. Firstly, the tumor was found in a patient with a non-diseased liver, whereas in contrast, c-HCC-CCs develop in a background of chronic liver disease. Secondly, there was no organized pattern of tumor cells, such as strands or trabeculae of intermediate tumor cells, which usually appear as a background of marked desmoplastic stroma in c-HCC-CCs. Thirdly, the tumor had a rich vascularization and exhibited an unusual perivascular pseudorosette formation of tumor cells. Lastly, the immunophenotype of tumor cells as C-KIT^+^/EpCAM^+^/E-cadherin^+^/K7^−^/K19^−^/CD56^−^/CD133^−^ did not fit into any description of putative stem cell tumors of the liver.

The main differential diagnosis according to the World Health Organization classification was a subtype of c-HCC-CC with stem cell features and an intermediate cell subtype (4), corresponding with tumors previously described as primary liver carcinomas of intermediate (hepatocyte-cholangiocyte) phenotype (15). Although C-KIT is frequently expressed in intermediate cells of c-HCC-CCs with stem cell features, the intermediate cell subtype of c-HCC-CC was distinct from that of the tumor observed in this study based on its morphological and IHC phenotype. The intermediate cell subtype of a c-HCC-CC usually consists of strands or trabeculae of small, uniform cells with scant cytoplasms and hyperchromatic nuclei embedded within a desmoplastic stroma (4,7,15). Tumor cells with strands or trabeculae were not observed, with the exception of tumor cells infiltrating into the capsule and adjacent liver tissue. In addition, a desmoplastic reaction was absent in the tumor. Tumor cells of the intermediate cell subtype of c-HCC-CCs express hepatocytic markers (AFP or HepPar-1), biliary markers (K7 or K19) and/or putative stem cell markers (CD56, CD133 or EpCAM) (4,7,15). By contrast, the tumor cells in the tumor described were negative for AFP, HepPar-1, K7, K19, CD34, CD56 and CD133, and positive for C-KIT and EpCAM. As described, the majority of c-HCC-CCs develop in a background of chronic liver disease (4,7,15). Thus, these findings suggested that the tumor observed in the present case study was distinct from the intermediate cell subtype of c-HCC-CCs with stem cell features.

Another important differential diagnosis for the presented tumor was small-cell undifferentiated hepatoblastomas (SCUD-HBs), indicative of undifferentiated blastema cells. This type of HB is composed of poorly cohesive sheets of small cells resembling cells of neuroblastomas and small-blue-cell tumors (4). SCUD-HBs exhibit numerous mitotic figures, necrosis, and abundant apoptosis. Although very few studies have analyzed the SCUD immunophenotype, SCUD cells have been shown to be positive for intermediate filaments typical of epithelial and mesenchymal cells, with positive expression for keratin and vimentin, rarely expressing CD99 and negative for AFP expression (4). SCUD-HB is considered to be a high-risk malignant tumor with poor prognosis, which reflects its high proliferative activity. In contrast to SCUD-HB, the present tumor exhibited low proliferative activity and a low-grade malignant potential. SCUD-HB is recognized in a subset of pediatric HBs, but has not been reported in adults (16). It remains unclear whether the present tumor represented a distinct disease entity with unique pathological features, or was a phenotypic variant of adult-type SCUD-HB.

Hepatic adult stem cells (HASCs) reside in portal areas within the Canals of Hering. Activation of HASCs in chronic liver disease leads to proliferation of bipotential transient amplifying cells or bipotential hepatic progenitor cells (HPCs), which can generate both hepatocytes and cholangiocytes (17). Tumors exhibiting features of cancer stem cells, including subsets of HCC, CC and c-HCC-CCs, may originate from transformed bipotential HPCs (4–9,15). The cells of the tumor in the present study were positive for C-KIT, EpCAM and E-cadherin, but negative for other known HPC markers including AFP, K19, CD34, CD56, CD133 and albumin. Badve *et al* (9) reported that undifferentiated small cell components in HB do not express HepPar-1, CD34 or K19, and proposed the possible existence of more primitive and undifferentiated progenitor cells in the liver. Similarly, Fiegel *et al* (8) identified primitive stem cells within connective tissue in human HBs positive for C-KIT but negative for all other markers tested (CD34, Thy1, K18, K7 and CD56). It was suggested that different types of stem cells may be present during histogenesis of HB (8). Based on these observations, in combination with the undifferentiated morphology of the tumor cells observed in the present study, it was hypothesized that the tumor originated from more primitive and undifferentiated cells rather than from bipotential HPCs. This notion was supported by data showing that a significant proportion of definite endodermal (DE) cells derived from embryonic stem (ES) cells on day 5 were positive for C-KIT and/or E-cadherin (18). C-KIT and E-cadherin have been used as representative surface markers in combination with CXCR4 to define ES cell-derived DE cells, and EpCAM expression has been shown to be expressed in ES, DE and HPC cells (18,19). During hepatic differentiation of DE cells, EpCAM-positive cells constitute a substantial proportion of the total cell population between days 5 and 13, whereas few C-KIT-positive cells have been identified on day 13, suggesting that the abundance of C-KIT-positive cells progressively decreases during ES cell differentiation (18). Cell populations that are C-KIT^−^/EpCAM^+^ have been demonstrated to be ES cell-derived hepatoblast-like progenitor cells based on morphological characterization and expression of hepatoblast-specific genes including AFP, albumin, K18 and K19 (18). Overall, the C-KIT^+^/EpCAM^+^/E-cadherin^+^/K7^−^/K19^−^/AFP^−^/albumin^−^ immunotype suggested that the present tumor may have originated from transformed C-KIT^+^/EpCAM^+^ DE-like cells, which are more primitive and undifferentiated than bipotential HPCs.

To the best of our knowledge, similar lesions have not been described previously in the literature and, therefore, this C-KIT-positive undifferentiated tumor may represent a previously unrecognized distinct tumor type of the liver. Further studies with a larger number of cases will be necessary to characterize the phenotype and nature of this undifferentiated hepatic tumor type.

## Figures and Tables

**Table I:** Summary of immunohistochemical results: Expression results of applied antibodies.

Immunoreactive antibodies	Positive cells (%)	Intensity	Staining pattern
C-KIT	>95	3+	Cytoplasmic dot, cell membrane accentuation
EpCAM	30	3+	Peripheral portion of tumor, cell membrane
EMA	30	3+	Peripheral portion of tumor, cytoplasm
E-cadherin	>95	3+	Cell membrane, occasional cytoplasm
β-catenin	>95	3+	Cell membrane, no nuclear translocation
S100 protein	20	2+	Nuclear and cytoplasm
α1-AT	30	3+	Cytoplasm and nuclear
α1-ACT	30	3+	Cytoplasm and nuclear
Pankeratin	5	2+	Cytoplasm
Keratin 7	<1	1+	Cytoplasm
Keratin 19	<1	1+	Cytoplasm
TP53	10	2+	Nuclear

Non-immunoreactive antibodies included: CD34, leukocyte common antigen, vimentin, CD99, HMB45, HepPar1, chromogranin, synaptophysin, TTF-1, CD56, platelet-derived growth factor receptor-alpha, CD133, albumin and α-fetoprotein. EMA, epithelial membrane antigen; α1-AT, α1-antitrypsin; α1-ACT, α1-antichymotrypsin.
